# Supplementary material for: Structural Characterization of Pinnatoxin Isomers
Source: Mar Drugs. 2025 Feb 26;23(3):103. doi: 10.3390/md23030103 (PMC11944192; doi:10.3390/md23030103)
Supplement: Supplementary file 1 [file marinedrugs-23-00103-s001.zip › marinedrugs-3478875-supplementary.pdf]

Structural characterization of pinnatoxin isomers

Andrew I. Selwood, Christopher O. Miles, Alistair L. Wilkins, Sarah C. Finch and Roel van Ginkel

		Page
Figure S1	^{1}H NMR spectrum of isopinnatoxin E (**5**)	S2
Figure S2	COSY NMR spectrum of isopinnatoxin E (**5**)	S3
Figure S3	TOCSY NMR spectrum of isopinnatoxin E (**5**)	S4
Figure S4	HSQC NMR spectrum of isopinnatoxin E (**5**)	S5
Figure S5	HMBC NMR spectrum of isopinnatoxin E (**5**)	S6
Figure S6	NOESY NMR spectrum of isopinnatoxin E (**5**)	S7
Figure S7	APT ^{13}C NMR spectrum of isopinnatoxin E (**5**)	S8
Figure S8	Comparison of ^{13}C NMR chemical shifts of **5** with those of **1** and **10**	S9
Figure S9	Structurally significant NOESY correlations of isopinnatoxin E (**5**)	S10
Figure S10	Isomerization kinetics for pinnatoxin E (**1**)	S11
Figure S11	Isomerization kinetics for pinnatoxin F (**2**)	S12
Figure S12	Isomerization kinetics for pinnatoxin D (**3**)	S13
Figure S13	Isomerization kinetics for pinnatoxin H (**4**)	S14
Figure S14	Isomerization kinetics for pinnatoxin G (**9**)	S15
Figure S15	Isomerization equilibration reaction profiles for pinnatoxins **1**, **2** and **5**	S16
Table S1	Body and organ weights of necropsied mice	S17

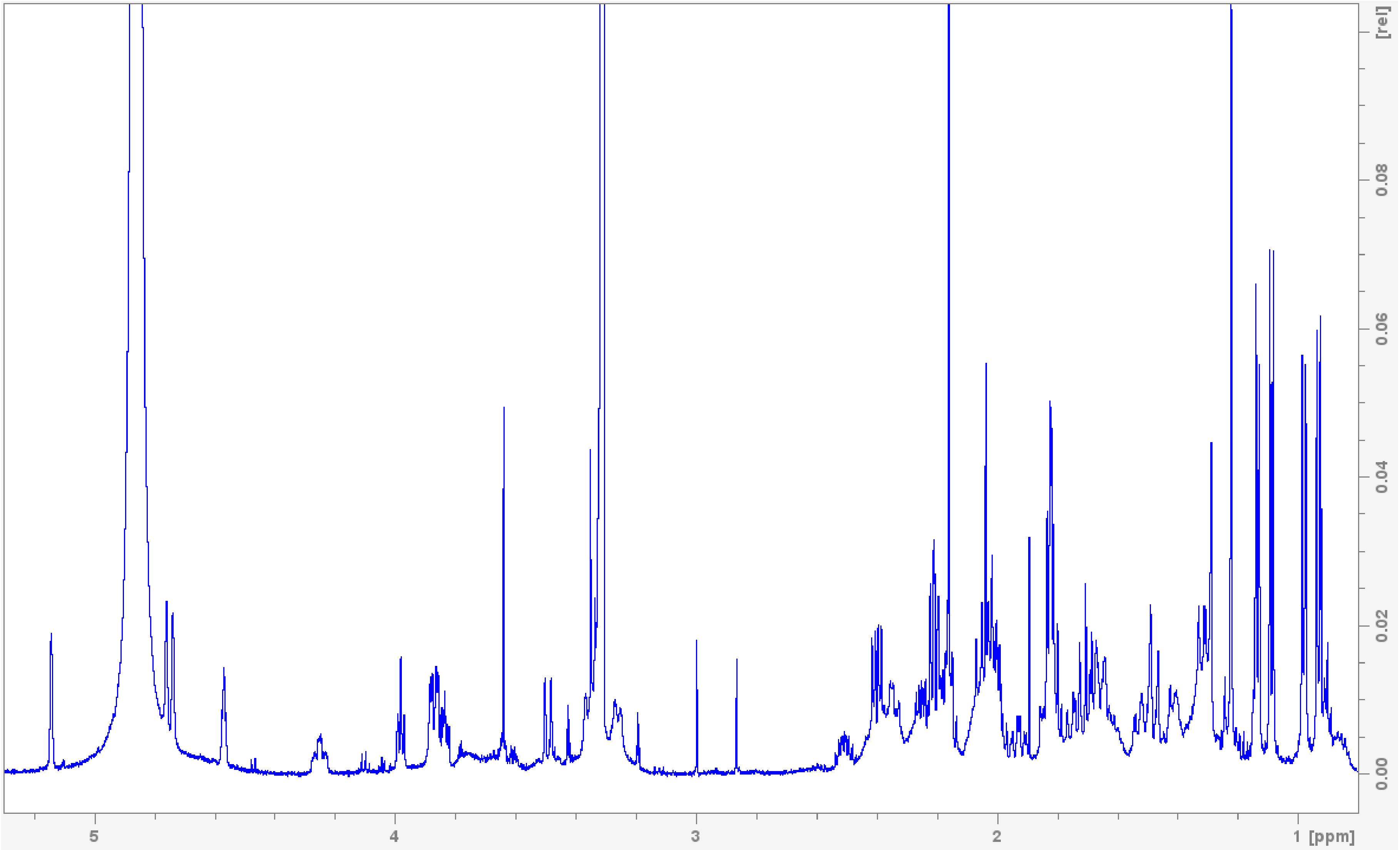

Figure S1. ^{1}H NMR spectrum of isopinnatoxin E (**5**) in CD_3OD.

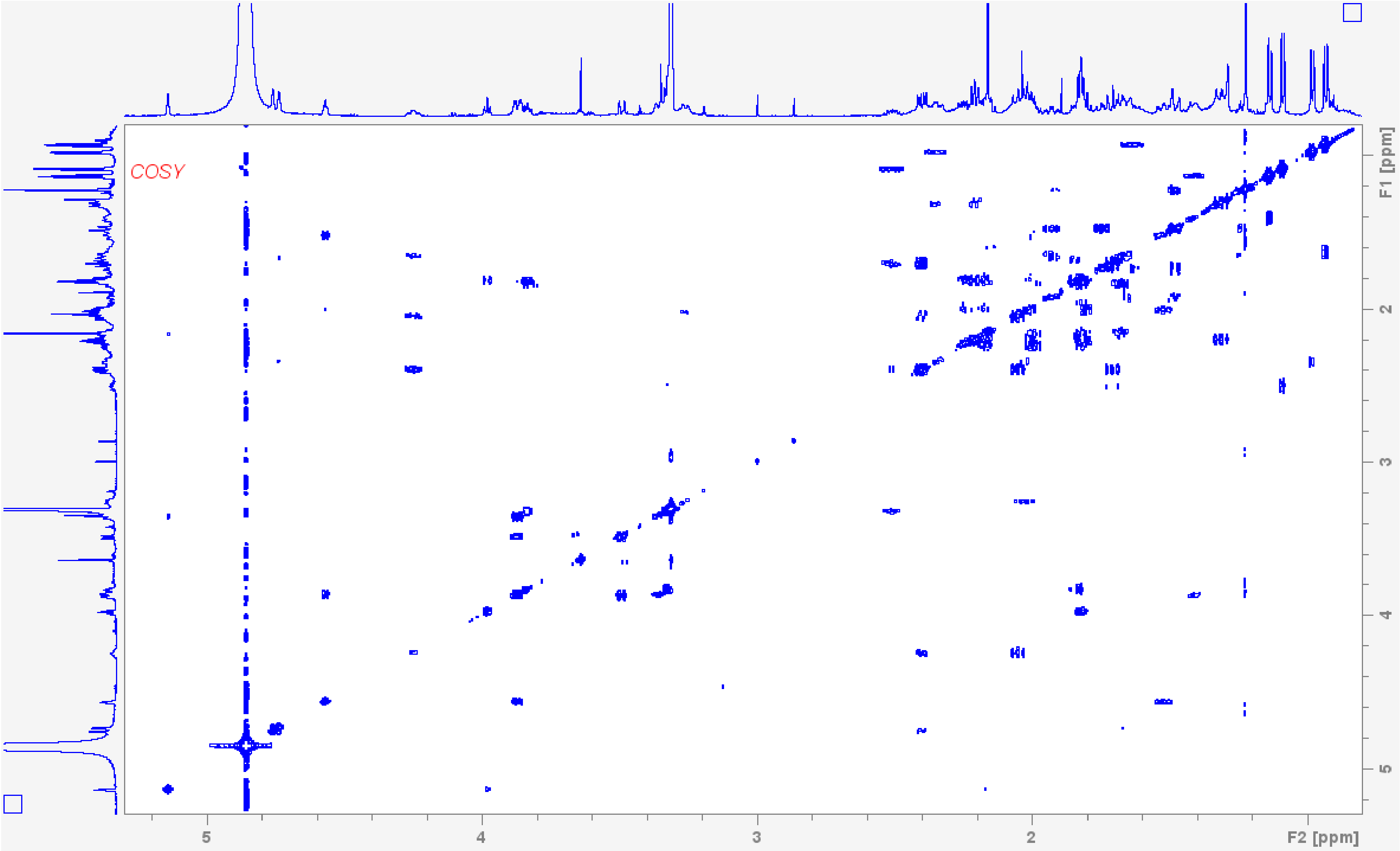

Figure S2. COSY NMR spectrum of isopinnatoxin E (**5**) in CD_3OD.

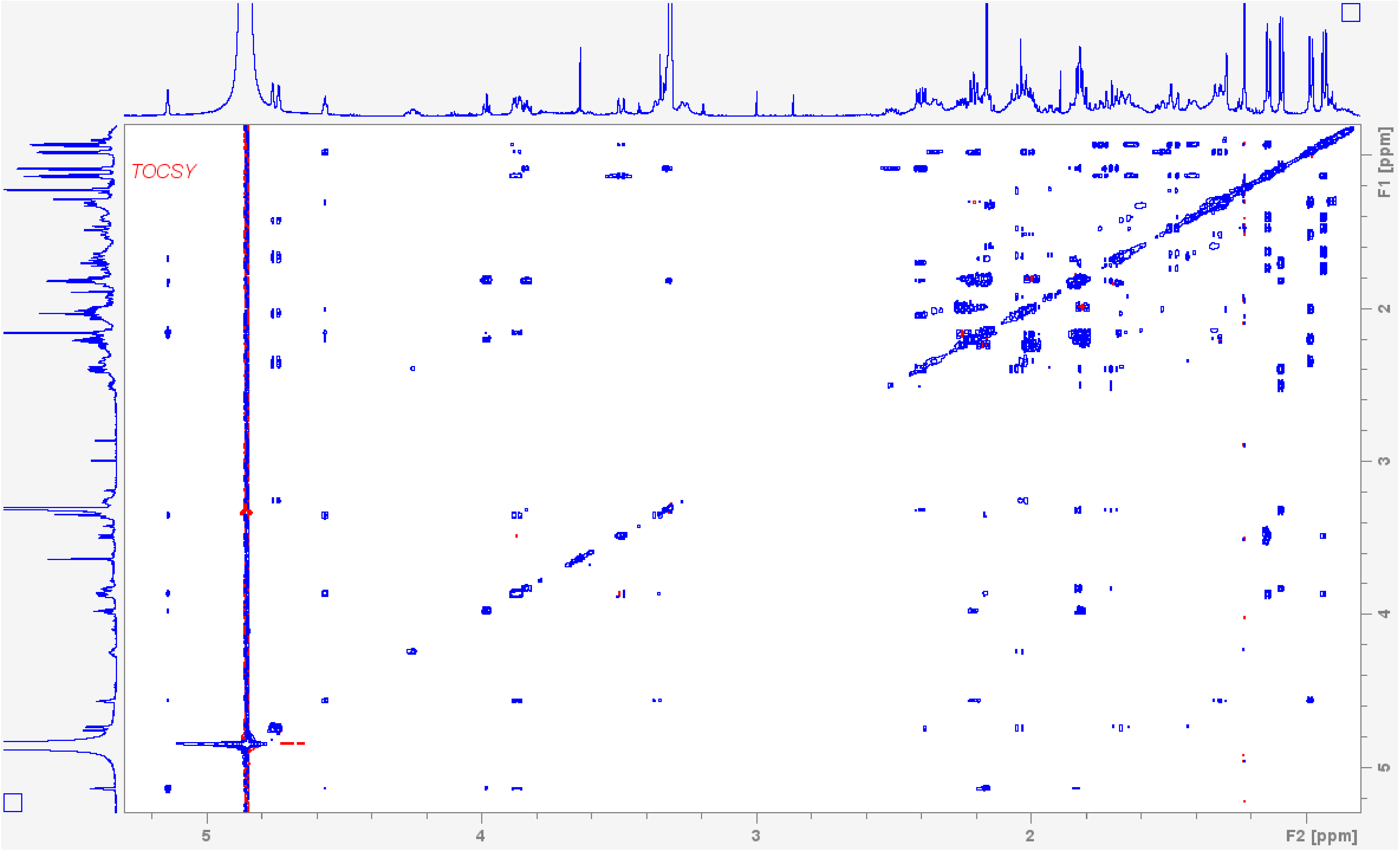

Figure S3. TOCSY NMR spectrum (160 ms) of isopinnatoxin E (**5**) in CD_3OD.

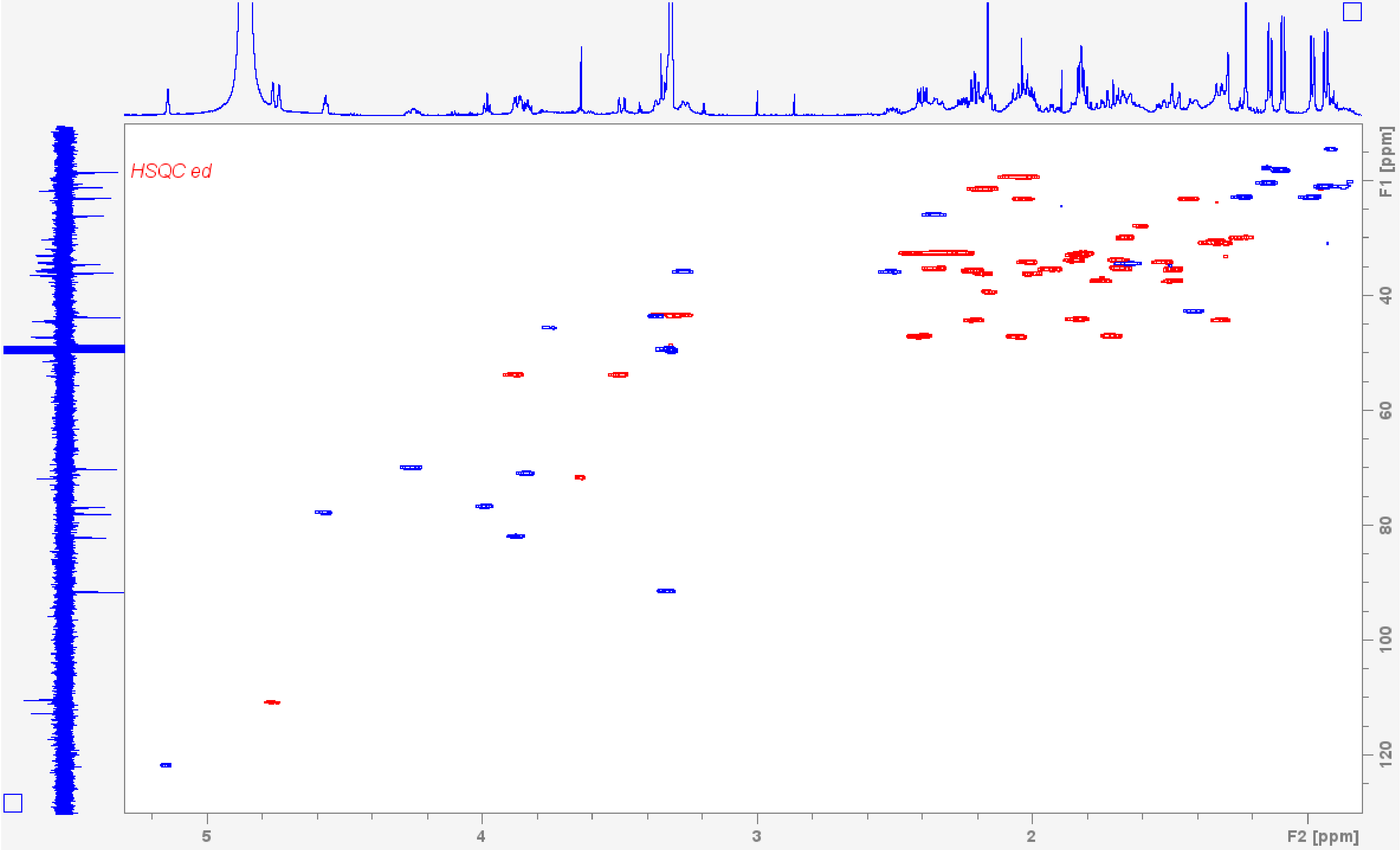

Figure S4. Multiplicity-edited HSQC NMR spectrum of isopinnatoxin E (**5**) in CD_3OD.

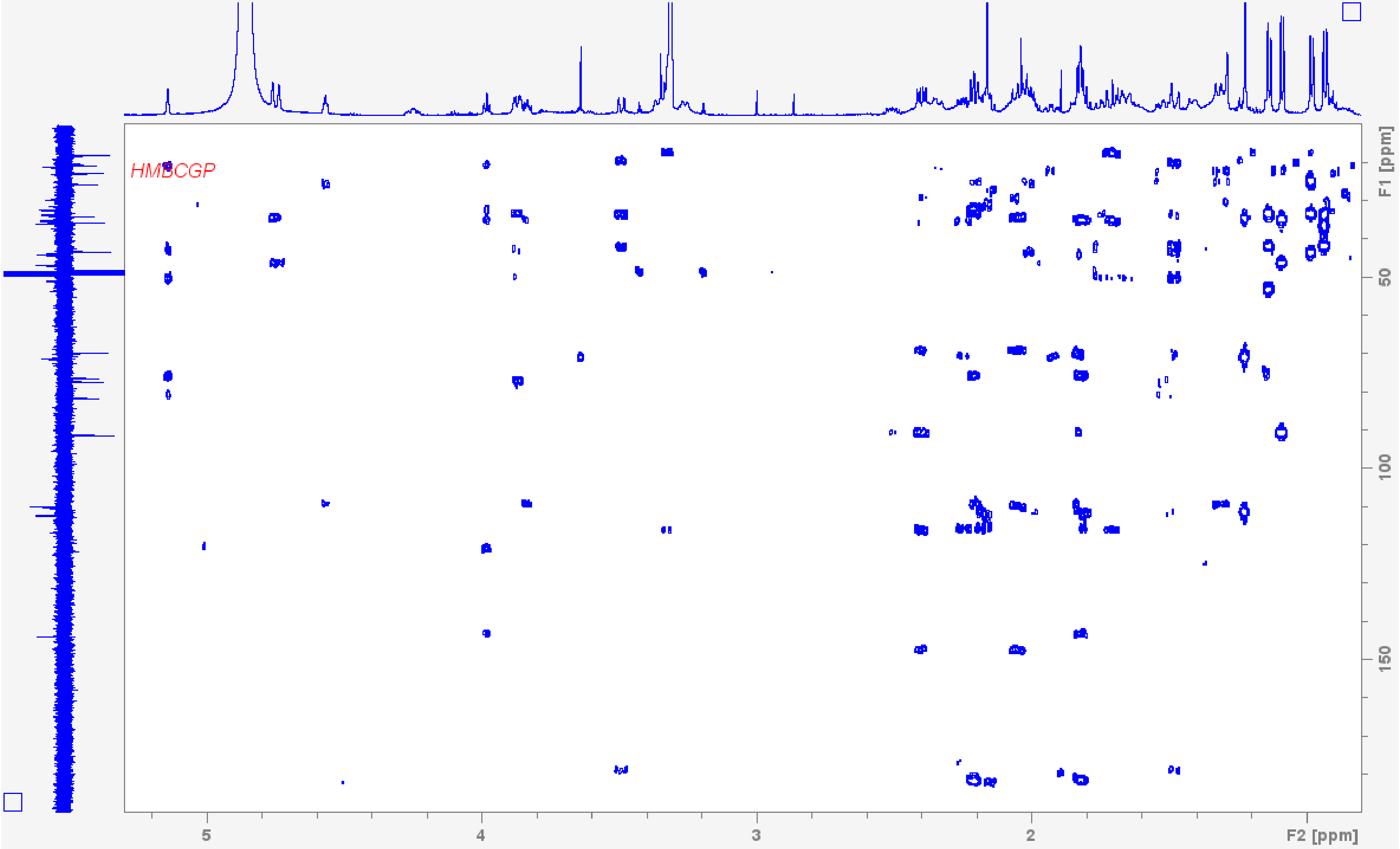

Figure S5. HMBC NMR spectrum of isopinnatoxin E (**5**) in CD_3OD.

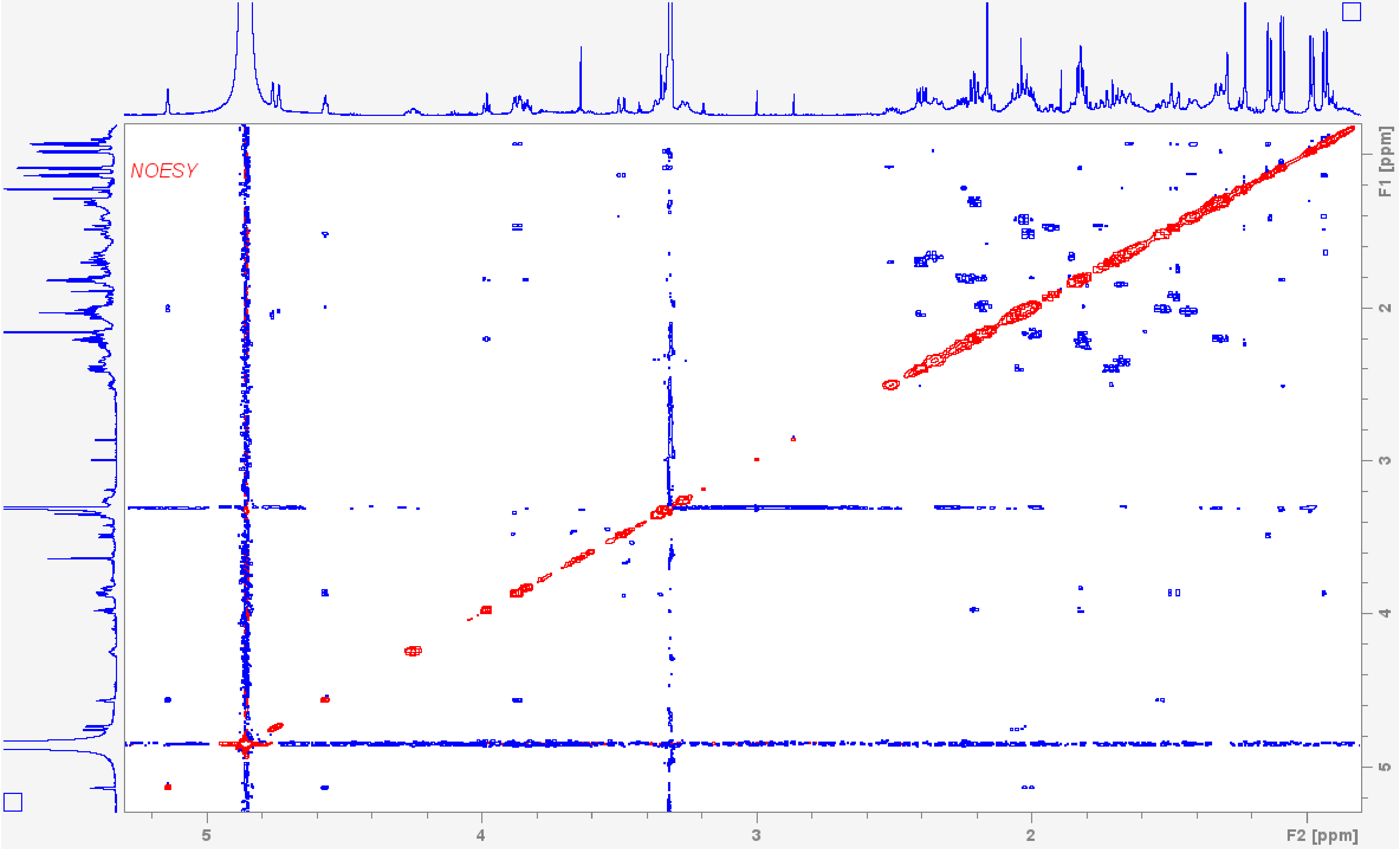

Figure S6. NOESY NMR spectrum of isopinnatoxin E (**5**) in CD_3OD.

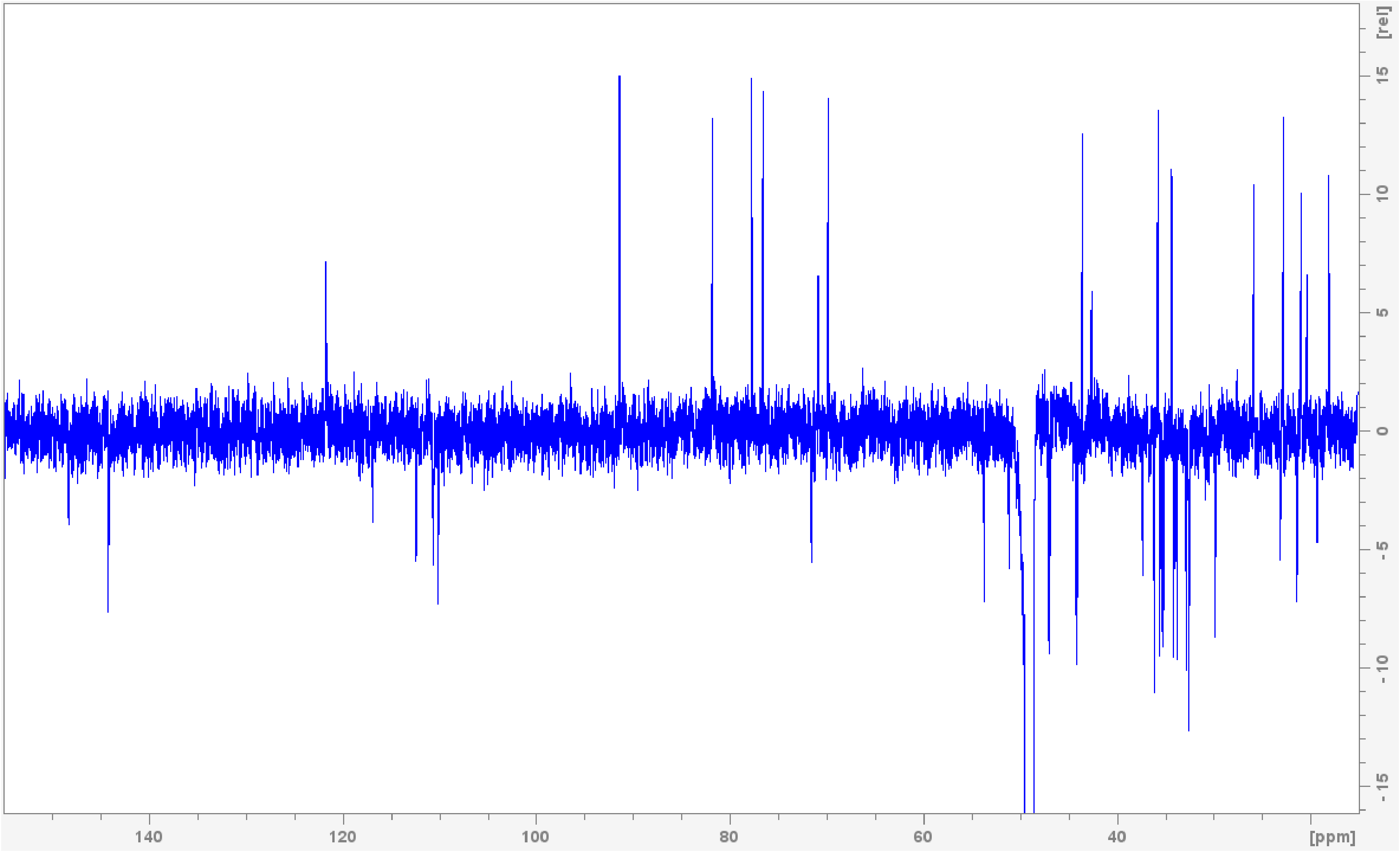

Figure S7. ^{13}C APT NMR spectrum of isopinnatoxin E (**5**) in CD_3OD.

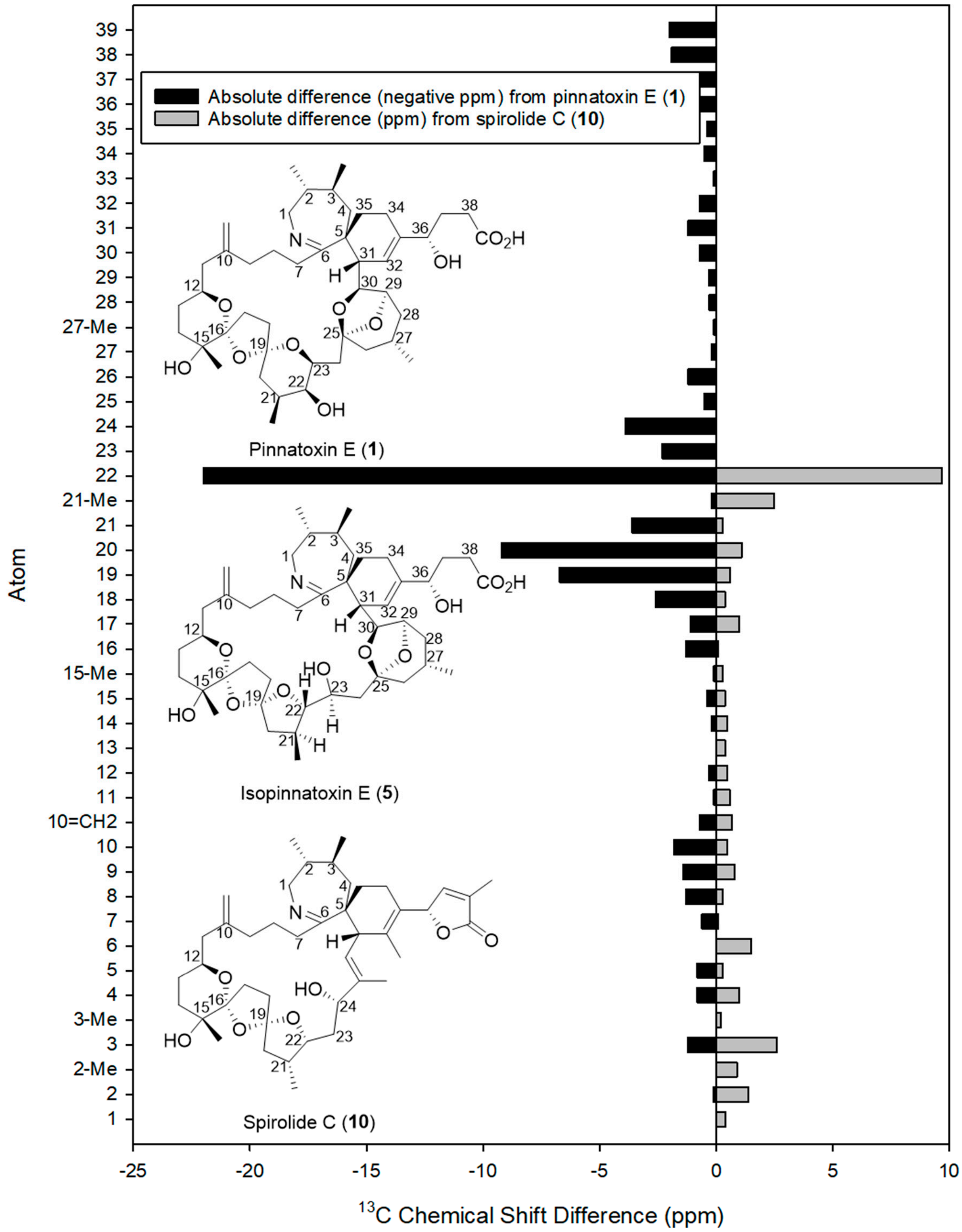

Figure S8. Absolute values of the ^{13}C NMR chemical shift differences between isopinnatoxin E (**5**) and the equivalent atoms and their substituents in pinnatoxin E (**1**) (except for C-6, which was not detected for pinnatoxin E), and in spirolide C (**10**) (from C-1 to C-22 only). Note the major perturbations to ^{13}C chemical shifts in the C- (C-16–C-19), and especially D-rings (C-19–C-22) of isopinnatoxin E (**5**) relative to those of pinnatoxin E (**1**).

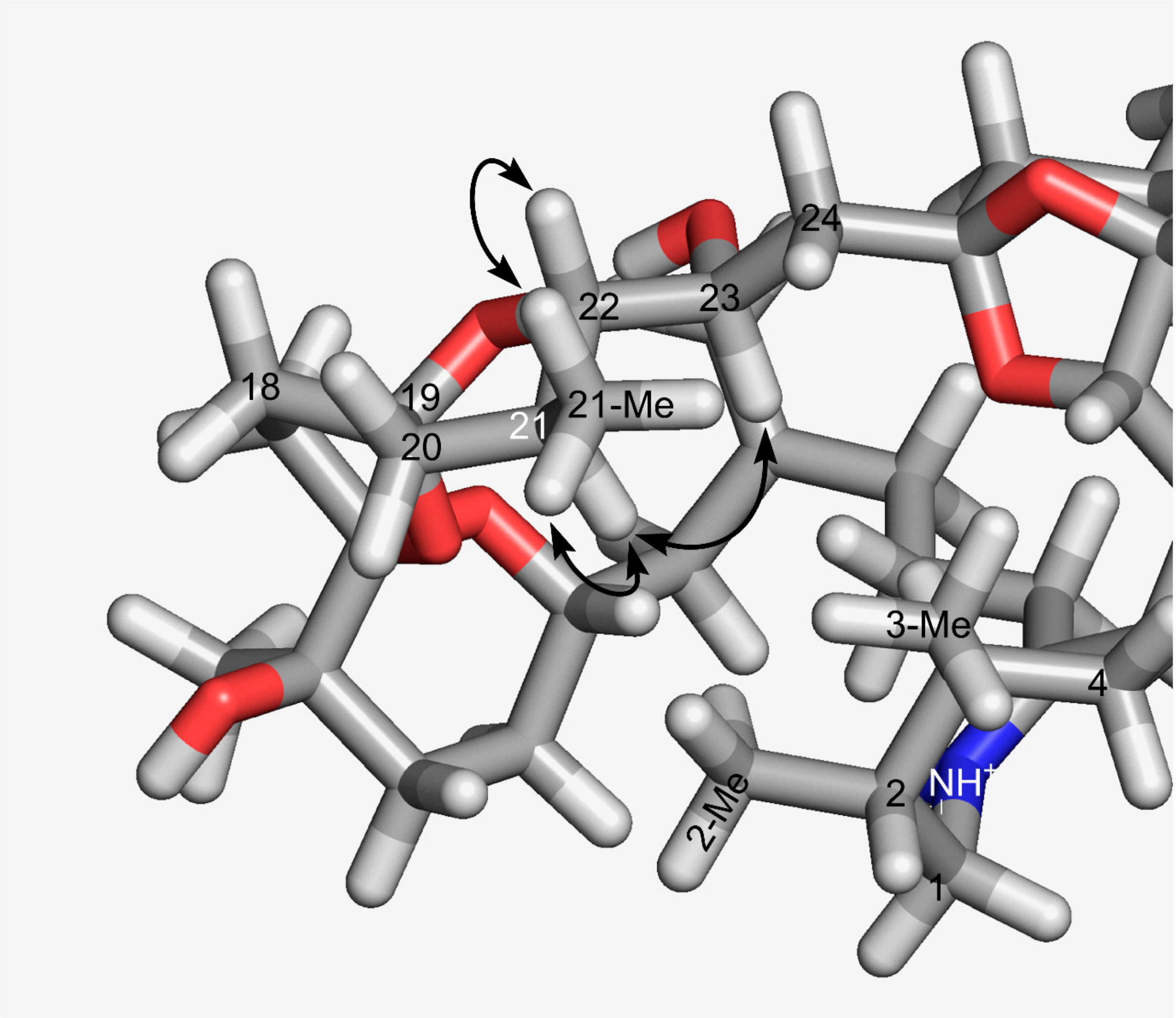

Figure S9. Molecular model of isopinnatoxin E (**5**) showing the structurally significant NOESY correlations (black arrows) in the C-21 to C-23, where acid-catalyzed isomerization of the tetrahydropyranyl D-ring of pinnatoxin E (**1**) has resulted in a tetrahydrofuranyl D-ring in **5**. The dihedral angles between H-23 and the H-21 and the two H-24 methylene protons in the modelled structure are consistent with the multiplicity and coupling constants of H-23 (ddd, J = 8.2, 6.0, and 3.3 Hz) that were observed in the ^{1}H NMR spectrum of **5**.

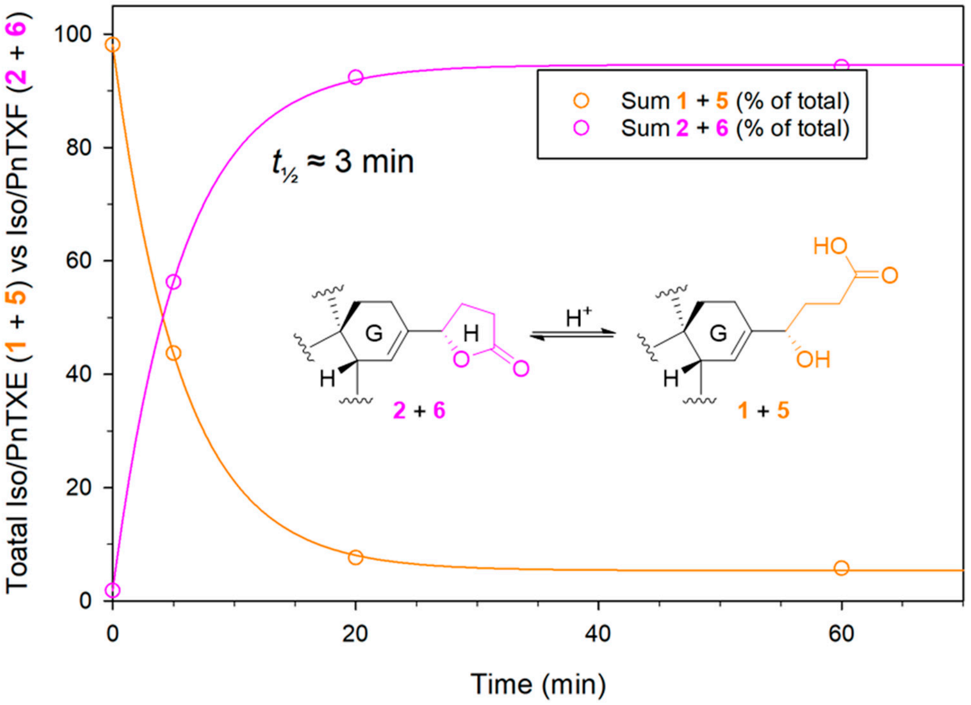

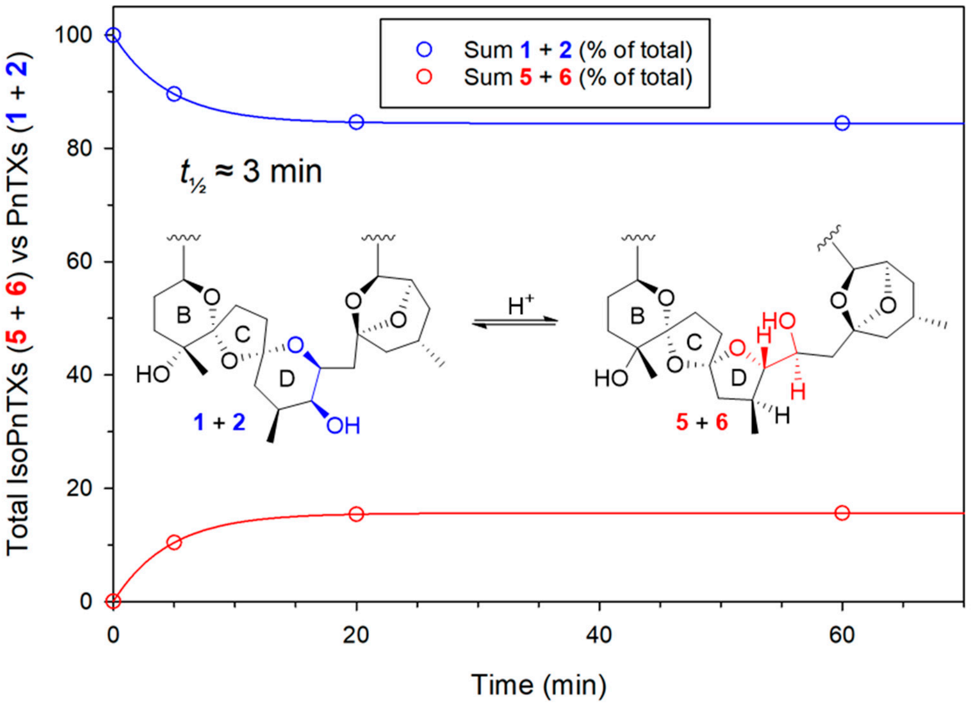

Figure S10. Mole fractions (%) of the four structural forms of pinnatoxin E (**1**) in the pinnatoxin (**1** and **2**) versus isopinnatoxin (**5** and **6**) forms, and in the lactone (**2** and **6**) versus γ-hydroxycarboxylic acid (**1** and **5**) forms, with time, obtained after addition of pinnatoxin E (**1**) to aqueous acetonitrile containing TFA. Data was fitted to 3-parameter exponential curves to provide equilibrium positions (Table 2) and half-lives for the isomerization reactions. Similar experiments were also performed to evaluate equilibration of **2**, **3**, **4**, **5**, and **9** (Table 2, Figures 4, S11–S14).

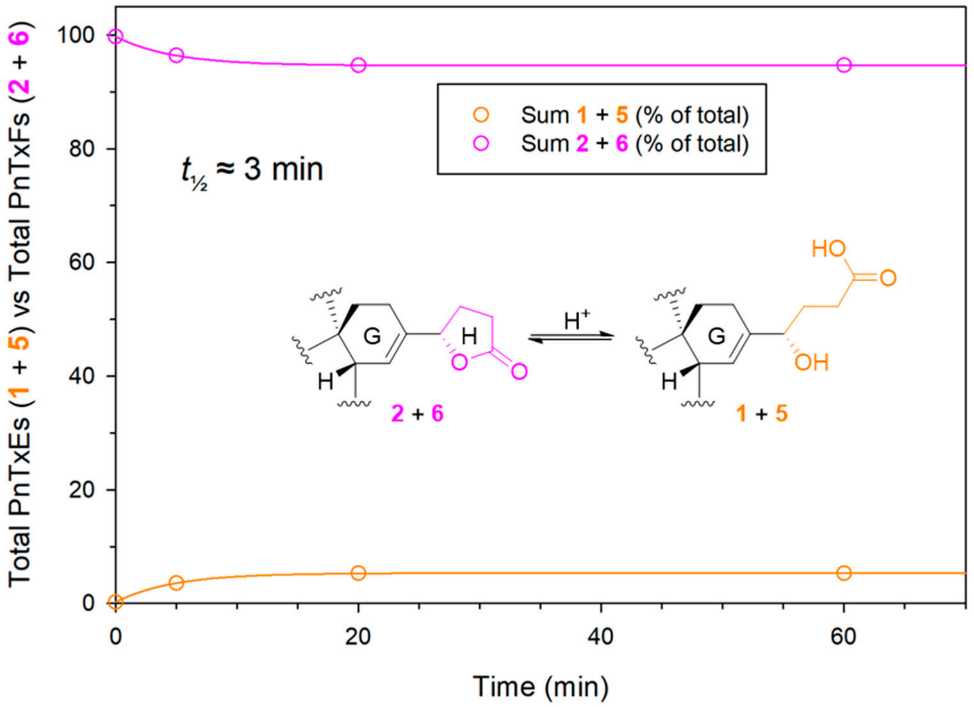

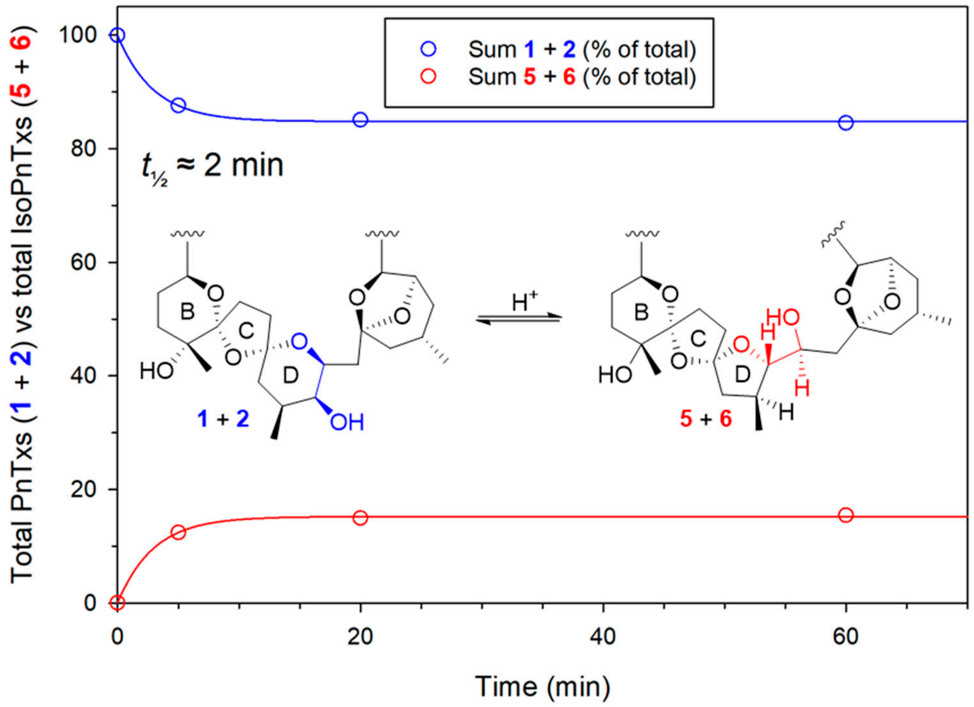

Figure S11. Mole fractions (%) of the four structural forms of pinnatoxin F (**2**) in the pinnatoxin (**1** and **2**) versus isopinnatoxin (**5** and **6**) forms, and in the lactone (**2** and **6**) versus γ-hydroxycarboxylic acid (**1** and **5**) forms, with time, obtained after addition of pinnatoxin F (**2**) to aqueous acetonitrile containing TFA. Data was fitted to 3-parameter exponential curves to provide equilibrium positions (Table 2) and half-lives for the isomerization reactions. Similar experiments were also performed to evaluate equilibration of **1**, **3**, **4**, **5**, and **9** (Table 2, Figures 4, S10, S12–S14).

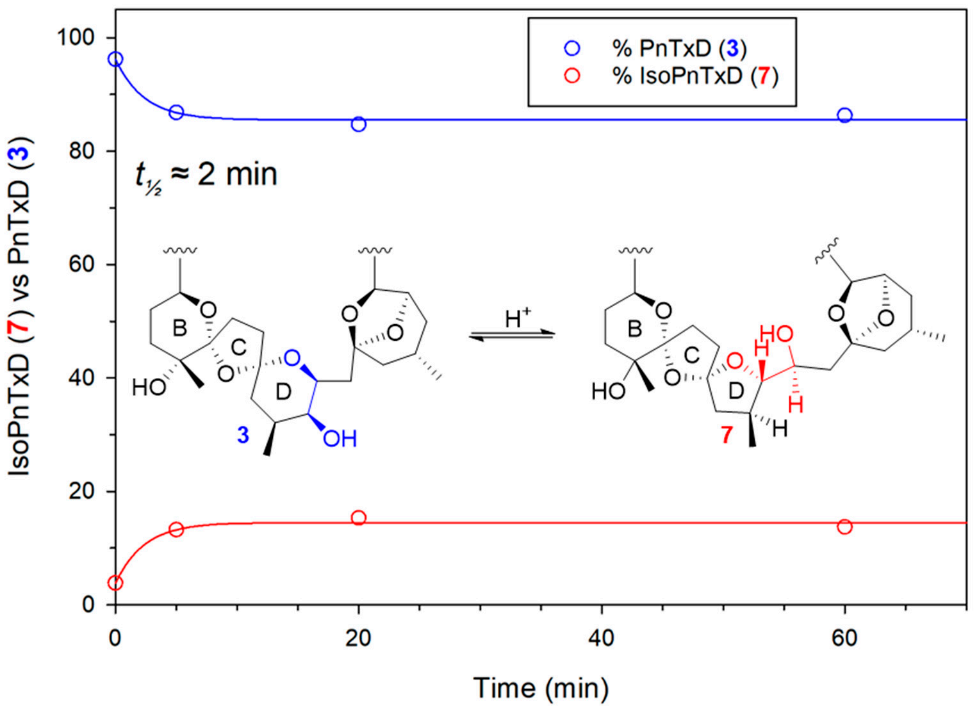

Figure S12. Mole fractions (%) of the two isomers of pinnatoxin D (**3**) in the pinnatoxin (**3**) versus isopinnatoxin (**7**) forms, with time, obtained after addition of pinnatoxin D (**3**) to aqueous acetonitrile containing TFA. Data was fitted to 3-parameter exponential curves to provide equilibrium positions (Table 2) and half-lives for the isomerization reaction. Similar experiments were also performed to evaluate equilibration of **1**, **2**, **4**, **5**, and **9** (Table 2, Figures 4, S10–S11, S13–S14).

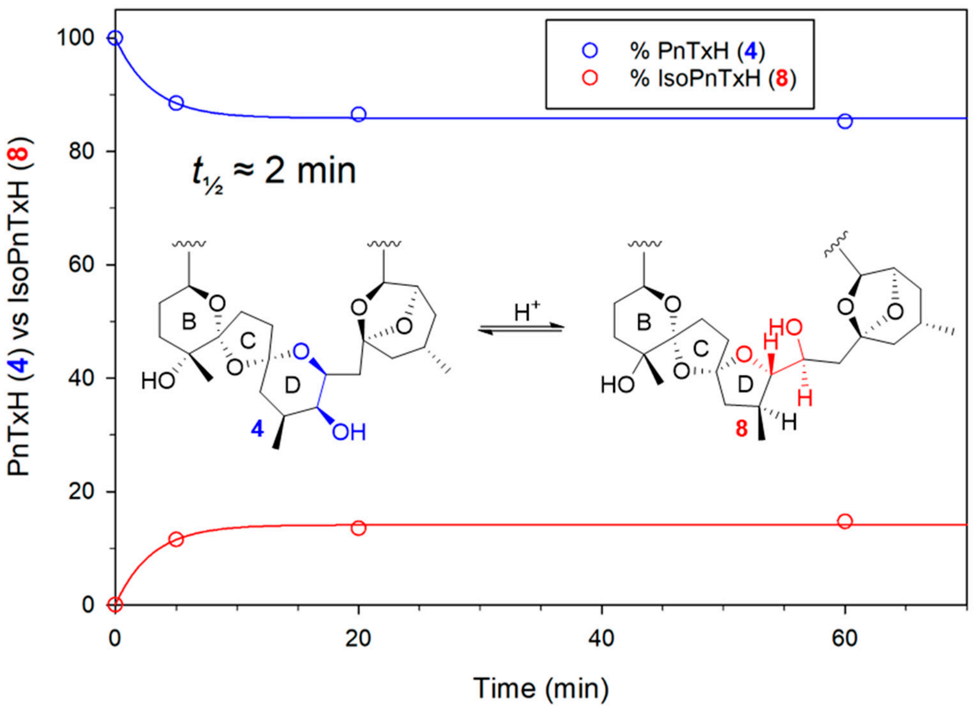

Figure S13. Mole fractions (%) of the two isomers of pinnatoxin H (**4**) in the pinnatoxin (**4**) versus isopinnatoxin (**8**) forms, with time, obtained after addition of pinnatoxin H (**4**) to aqueous acetonitrile containing TFA. Data was fitted to 3-parameter exponential curves to provide equilibrium positions (Table 2) and half-lives for the isomerization reaction. Similar experiments were also performed to evaluate equilibration of **1**, **2**, **3**, **5**, and **9** (Table 2, Figures 4, S10–S12, S14).

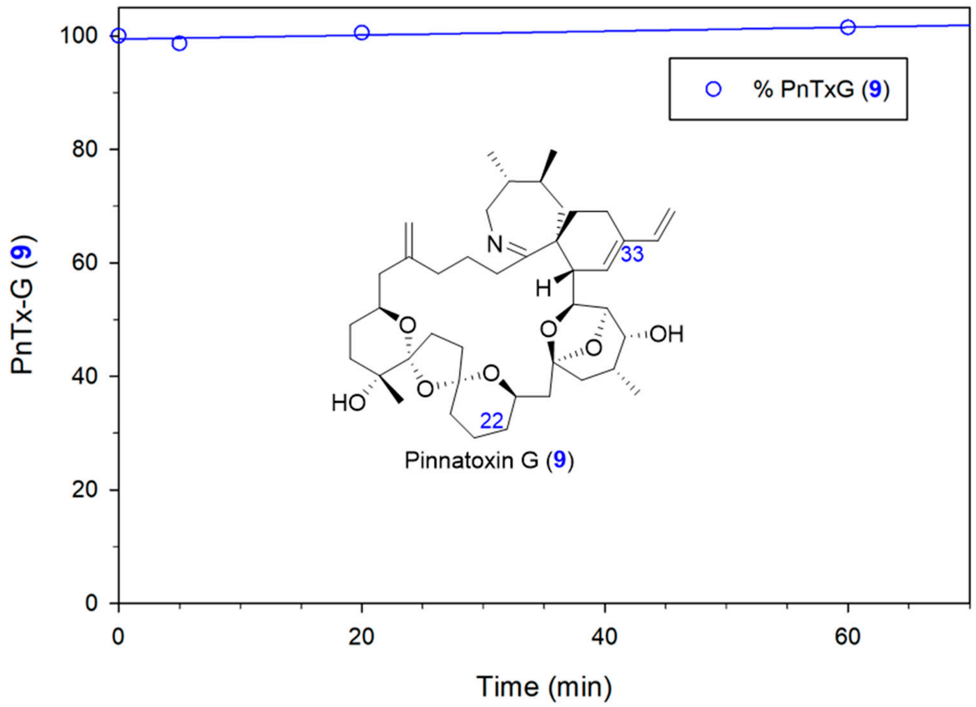

Figure S14. Mole fractions (%) of pinnatoxin G (**9**) in the pinnatoxin (**9**) with time, obtained after addition of pinnatoxin G (**9**) to aqueous acetonitrile containing TFA. Data was fitted to a straight line because no detectable equilibration was observed (Table 2). Similar experiments were also performed to evaluate equilibration of **1**, **2**, **3**, **4**, and **5** (Table 2, Figures 4, S10–S13).

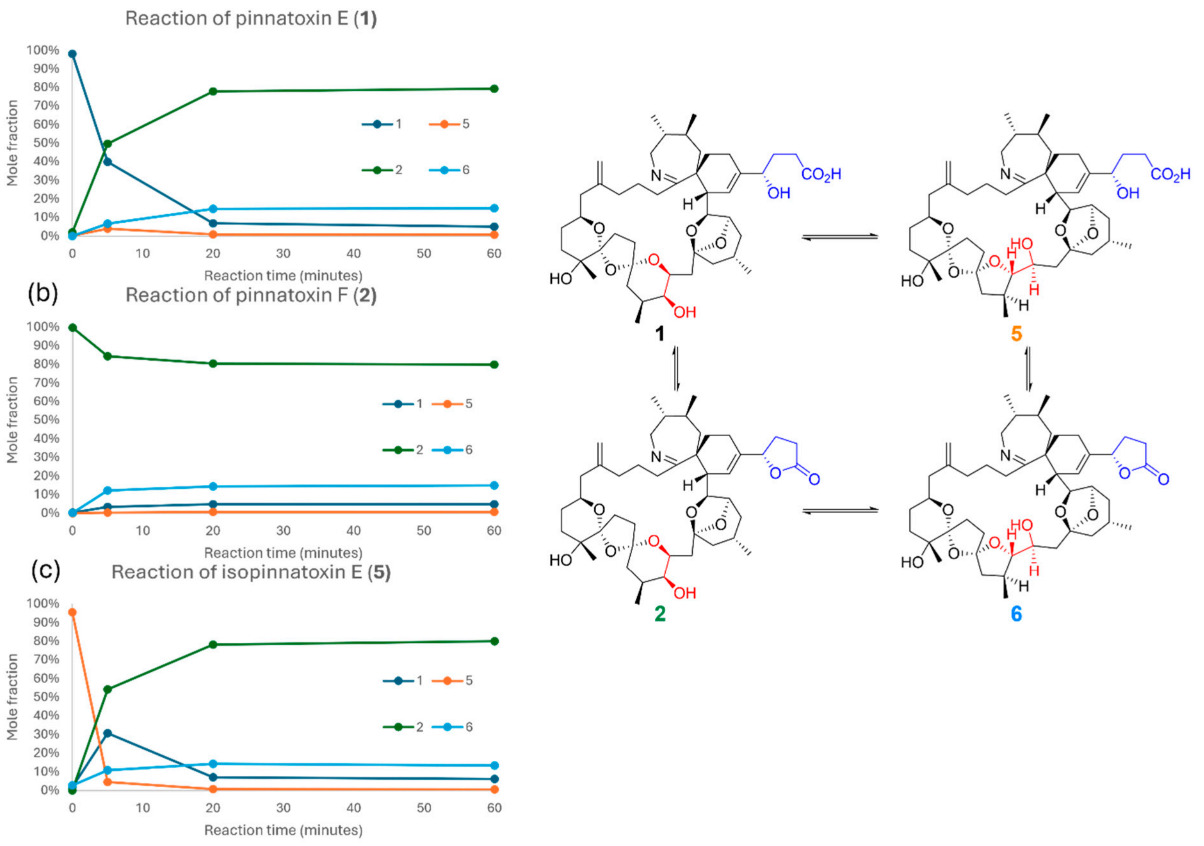

Figure S15. Time-courses of equilibration of pinnatoxin E derivatives: (a) **1**; (b) **2**, or; (b) **5** in acidic solution, to form mixtures of **1**, **2**, **5**, and **6**. The graphs show the mole fractions of each of the four equilibrating analogues with time, while the chemical sub-figure (d) shows the structures of the four analogues and their chemical interconversions via isomerization of their D-rings (red substructure) and opening and closing of their lactone moieties (blue substructure).

Table S1. Relative organ weights (% of body weight) of mice dosed with isopinnatoxin E (**5**)

**Organs**	**Dose levels µg/kg**					
	**280**	**400**	**700**	**980 (a)**	**980 (b)**	**980 (c)**
Liver	5.7%	5.1%	5.8%	5.2%	5.4%	5.1%
Kidneys	1.5%	1.4%	1.4%	1.4%	1.3%	1.5%
Spleen	0.71%	0.63%	0.68%	0.50%	0.41%	0.46%
Heart	0.55%	0.55%	0.56%	0.59%	0.54%	0.57%
Lungs	0.89%	0.92%	1.2%	1.8%	0.86%	1.1%
Full stomach	2.0%	2.2%	1.8%	2.3%	2.2%	1.8%
Empty stomach	0.79%	0.71%	0.74%	0.66%	0.66%	0.76%
Gut	11%	9.3%	12%	12%	13%	9.6%
